# Belfast Limb Arterial and Skeletal Trauma (BLAST): the evolution of punishment shooting in Northern Ireland

**DOI:** 10.1007/s11845-017-1561-8

**Published:** 2017-01-21

**Authors:** M. Lau, S. McCain, R. Baker, D. W. Harkin

**Affiliations:** Belfast Vascular Centre, Royal Victoria Hospital, Belfast, Northern Ireland

**Keywords:** Gunshot injury, Punishment shootings, Belfast, Popliteal fossa, Tourniquet, Intraluminal shunt, Civilian, BLAST

## Abstract

**Introduction:**

Northern Ireland has developed significant experience in specific punishment injuries due to its unique civil unrest. Simple gunshot wound (GSW) injuries have begun to evolve into more complex injuries.

**Case presentation:**

We describe three cases of young male victims who suffered from GSW injuries—from a single GSW injury to multiple GSW injuries involving all four limbs; the phenomenon of Belfast Limb Arterial and Skeletal Trauma. We describe the management of these injuries, with a review of current literature.

**Conclusion:**

Due to the unique political situation of Northern Ireland, there has been significant development of surgical experience in GSW management. Historically, single knee-capping injuries were prevalent. However, these shootings have evolved into targeted injuries resulting in significant trauma as demonstrated by this case series.

## Introduction

Northern Ireland, as a result of a period of prolonged civil unrest, often referred to as ‘the troubles’, has developed significant experience in the management of civilian GSW injuries [1, 2]. Perhaps, unique to our region was a phenomenon of punishment shootings by illegal organisations as a form of control within the civilian population. These frequently involved GSW to the popliteal region, often referred to as ‘knee-capping’ injuries. These can cause severe arterial and venous injuries, in addition to damage of surrounding nerves and skeletal elements. Whilst political settlements have led to a reduction in gun crime, over the last decade, we have witnessed a re-emergence of punishment shootings which have evolved beyond knee-capping, to a form of anatomically targeted arterial and skeletal trauma: the phenomenon we describe here as the Belfast Limb Arterial and Skeletal Trauma (BLAST). These GSW are specifically targeted by the perpetrators to disrupt major arteries and skeletal elements at multiple sites to produce devastating and often irreparable injuries.

We present a case series with review of the literature to describe the phenomenon of BLAST injuries in Northern Ireland.

## Case series

### Case 1: gunshot wound to single lower limb (knee capping)

**Fig. 1:**
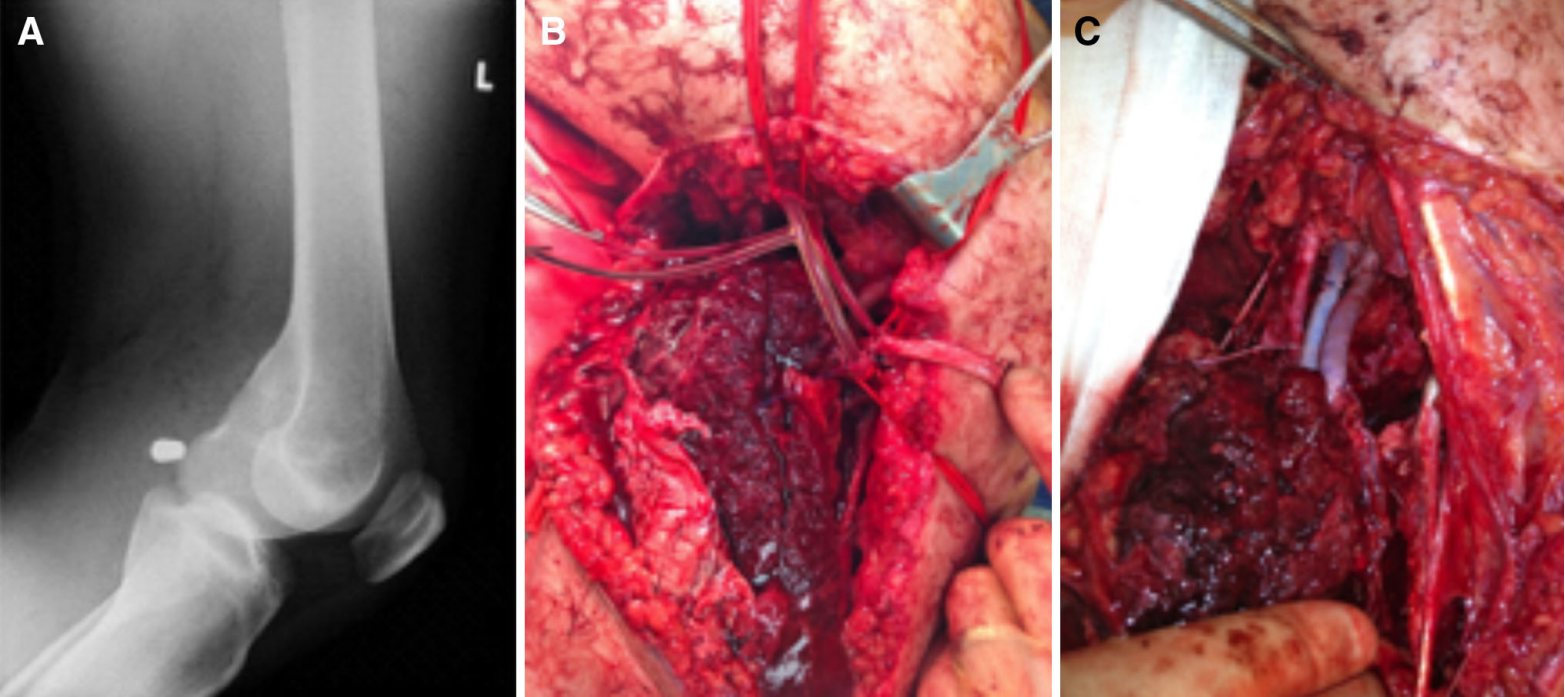
Popliteal GSW: knee-capping punishment injury

A 20-year-old male with a single GSW to his left lower limb, often referred to as *a* classical ‘knee-capping’ punishment shooting. The victim was invited under duress, by an illegal organisation to present himself to a planned outdoor location where he was subjected to a GSW to the left knee. Prior to his persecutors leaving the scene, they telephoned an emergency ambulance which transported him to a nearby Level 1 Trauma Centre. On arrival, he was noted to have a single entry wound in the left popliteal fossa, but no exit wound, and an expanding pulsatile haematoma at the site of injury, with evidence of external haemorrhage and acute limb ischaemia. He was resuscitated and had a plain radiograph of his left leg which demonstrated a low-velocity bullet foreign body in the popliteal fossa (Fig. 1). He was assessed by the vascular team and underwent emergency open exploration of the popliteal fossa via a medial below-knee approach. After control of the active haemorrhage, there was evidence of left popliteal arterial disruption and occlusion of the popliteal vein. A bullet was noted to be lodged in the popliteal fossa without damage to bone or nerve. He underwent intraluminal vascular shunting, in the popliteal artery and vein, and proceeded to an interposition autologous (harvested contralateral long saphenous vein) reversed vein graft repair of left popliteal artery and vein, Fig. 1. He made an uncomplicated recovery and regained good functional status.

### Case 2: gunshot wounds to both lower limbs (BLAST injury)

**Fig. 2:**
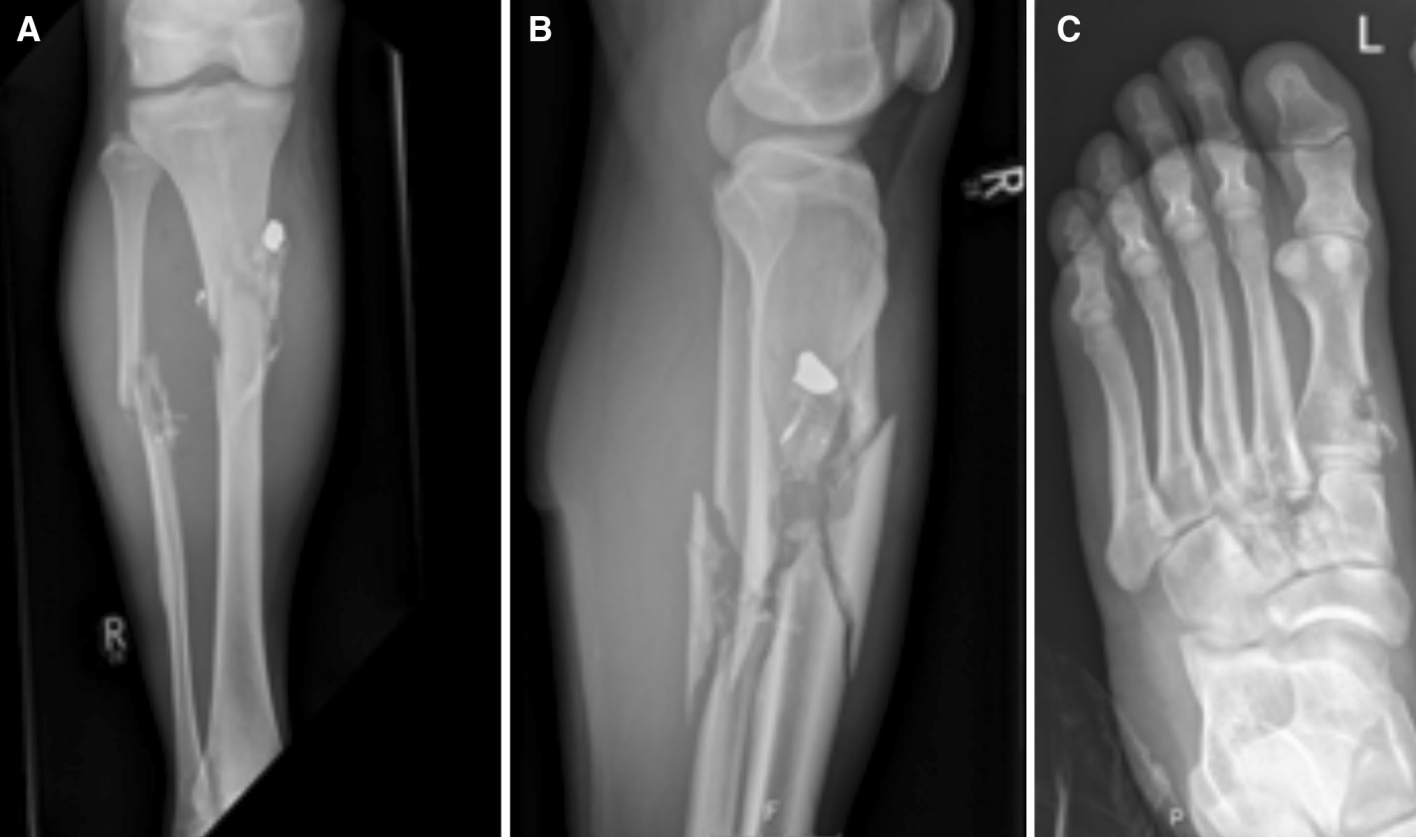
Belfast Limb Arterial and Skeletal Trauma (BLAST). BLAST victim with gunshot wounds to both lower limbs, including: *right lower limb* GSW with arterial trauma (anterior tibial artery and tibio-peroneal trunk), Skeletal trauma to fibula and tibia, and retained bullet (**a**, **b**), and *left lower limb* GSW with arterial trauma (dorsalis pedis artery) and skeletal trauma to first meta-tarsal bone (**c**)

A 20-year-old male was subjected to a home invasion by members of an illegal organisation and suffered multiple targeted GSW to both lower legs. Whilst restrained, he suffered a single GSW to the medial aspect of the right leg (below the knee) and a single GSW to the dorsum of the left foot. An emergency ambulance was requested by his relatives, after the departure of the persecutors, which transported him to a nearby Level 1 Trauma Centre. On arrival, he was noted to have a single entry wound to his right leg laterally at the midpoint of the fibula with supra-medial trajectory but no exit wound, and an expanding pulsatile haematoma medially below knee and acute limb ischaemia. He had a “through-and-through” GSW with an entry wound on the medial aspect of the dorsum of the left foot and an exit wound on the plantar surface. On examination, external haemorrhage had ceased and the vascularity of the left foot was satisfactory. He was initially resuscitated and underwent a plain radiograph of both legs, which demonstrated right fibula and tibia injury and a retained deformed bullet foreign body, in addition to skeletal trauma to the left first meta-tarsal bone (Fig. 2). He underwent a vascular assessment and proceeded to have an emergency open exploration of the right leg arteries below knee via a medial approach. When haemostasis was achieved, there was evidence of disruption of the right popliteal artery and tibio-peroneal (TP) trunk. A fragmented bullet was lodged with open comminuted fracture of the right fibula and tibia. He had intraluminal vascular shunts placed from the popliteal artery to TP trunk, and underwent short segment bypass from the popliteal artery to the distal TP trunk using interposition autologous (harvested contralateral long saphenous vein) reversed vein graft repair. He had an on-table angiogram which demonstrated an occluded right anterior tibial artery (without bleeding) and a patent bypass with run-off via the posterior tibial artery to the foot. We elected to manage his left foot conservatively as the bleeding had stopped, and despite occlusion of the left dorsalis pedis artery, his foot was well-collateralised and vascularly intact. He was also assessed by the Orthopaedic Surgeons, and had an external fixator to his right tibia. He made an uncomplicated recovery and regained good functional status.

### Case 3: gunshot wounds to all four limbs (BLAST injury)

**Fig. 3:**
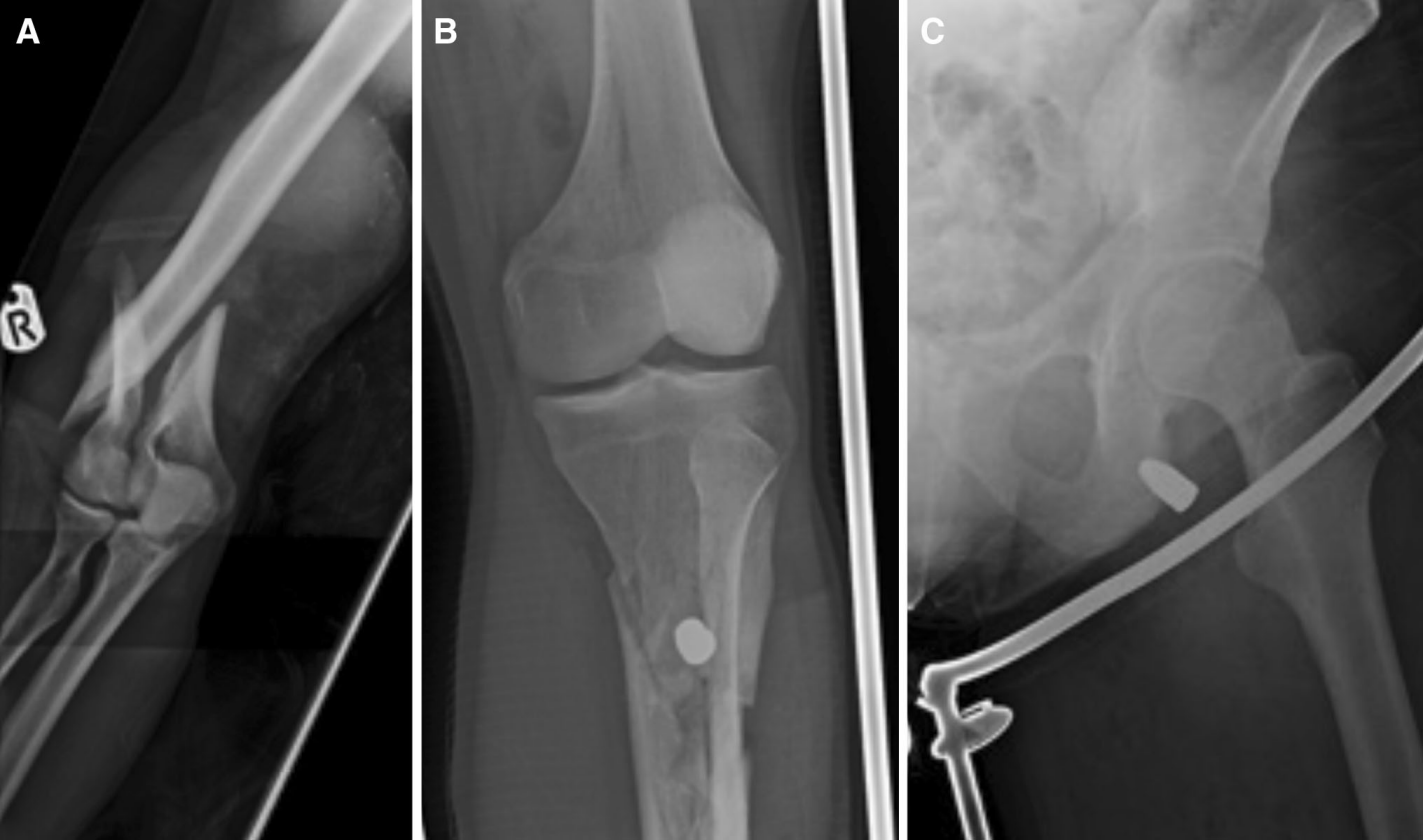
Belfast Limb Arterial and Skeletal Trauma (BLAST) BLAST victim with gunshot wounds to all four limbs (*both upper limbs* and *both lower limbs*). Displaced supracondylar fracture (**a**), comminuted tibial fracture (**b**), and gunshot injury to left ischium (**c**)

A 30-year-old male presented with GSW to all four limbs, after being subjected to home invasion by an illegal organisation. He was transported to a nearby district general hospital, via emergency ambulance, with a total of seven GSW. He was bleeding profusely from a wound in his left thigh and his left leg was acutely ischaemic. On arrival, he was haemodynamically unstable but responded to fluid resuscitation. Direct pressure failed to control the bleeding; initially, therefore, a tourniquet was applied. His initial imaging was in the form of plain radiographs which revealed a displaced right supracondylar fracture, bilateral tibia fractures, left femoral fracture, and fractured ischium of the pelvis, Fig. 3. He was transferred to a Level 1 Trauma Centre 60 miles away for further management and underwent a computed tomography angiogram (CTA) which confirmed the skeletal injuries and demonstrated injury to the left superficial femoral artery. An emergency exploration of his left thigh was performed by the vascular surgeons. He underwent an autologous vein patch to the left superficial femoral artery prior to external fixation of his humeral fracture and tibial fractures and had an intramedullary nail to his femoral fracture. Time from the incident to theatre was less than 4 h (including the ambulance transport). Prophylactic fasciotomies were not deemed necessary in this case. He suffered no short- or long-term complications associated with his vascular injury. He was discharged home and followed-up long-term under the orthopaedic team.

## Discussion

Northern Ireland, a devolved region of the United Kingdom and part of the island of Ireland, has been the focus of territorial and jurisdictional disputes since its inception in 1923. It has emerged from a prolonged period of civil unrest, often referred to as ‘the troubles’, during which paramilitary organisations conducted a campaign of violence [1]. One aspect of control exerted by such groups was the use of punishment beatings or shootings against the civilian population. Shootings commonly involved a GSW to the popliteal fossa, often referred to as ‘knee-capping’ injuries, which often caused severe arterial and venous injuries, in addition to damage of surrounding nerves and skeletal elements [1, 2]. The Northern Ireland experience in the management of these complex vascular injuries by vascular surgeons, in conjunction with multidisciplinary teams, including orthopaedic and plastic surgery specialists, allowed functional limb salvage in the majority of cases [2–4]. Lessons learned have helped in the management of civilian and battlefield vascular injuries worldwide. The culmination of years of political negotiation resulted in the “Good Friday” agreement in 1998, allowing decommissioning of arms by the major illegal organisations in Northern Ireland—which initially resulted in reduced gun crime. However, over the last decade, we have witnessed a re-emergence of punishment shootings which have evolved beyond knee-capping, to a form of anatomically targeted arterial and skeletal trauma: BLAST. We present a representative case series, demonstrating the evolution of punishment shooting and the spectrum of injury which can include one or two lower limbs, or at its most extreme all four limbs.

The principles of the management of BLAST injury are similar to that previously described for complex lower limb vascular injuries [1–4], and in extreme cases (multiple limbs) should consider modern damage-control surgical principles [5, 6]. The initial assessment and resuscitation should follow Advance Trauma and Life Support (ATLS) guidelines [7]. Volume resuscitation should be geared towards ‘permissive hypotension’ to allow adequate end-organ perfusion but avoids further blood loss [5]. Despite the varying degrees of trauma in the series described, each patient was initially resuscitated, prior to any surgical intervention. In severe BLAST injury (case 3, four limbs) with massive blood loss and haemodynamically instability, a tourniquet may help prevent exsanguination, especially if transport to a distant Trauma Centre is necessitated.

In all cases, timely assessment and recognition of vascular injuries will increase the chances of functional limb salvage [1–7]. Clinical diagnosis by an experienced clinician is essential, but this is often improved by judicious use of appropriate imaging. Plain radiographs are frequently performed in the emergency department and often give valuable information on skeletal elements and foreign bodies (bullets and missiles) during the early assessment and resuscitation phase. In the series described, useful information is demonstrated in all cases. Clinical assessment of pulses and adjuvant use of hand-held doppler are helpful but cannot be fully relied upon in the hypotensive patients or in those with complex injury mechanisms. Therefore, in stable patients, additional information can be rapidly obtained by CTA to diagnose vascular injuries and offer valuable information on skeletal and soft-tissue elements [8]. In those few patients too unstable for CTA, and with hard signs of vascular injury, damage-control surgery principles should be adopted with rapid transfer to the emergency theatre (or hybrid suite) for rapid control of haemorrhage by surgical and/or endovascular means with the guidance of on-table angiographic imaging [3–6]. Case 2 demonstrates the benefit of surgical control of bleeding with on-table angiography to assess multiple arterial injuries and allow informed decisions on active or conservative management of these. Hard signs of vascular injury include pain, pale and cold limb, pulsatile bleeding, expansile haematoma, paralysis, and absent distant pulses: Langer et al. found that these signs provide 92–95% sensitivity for predicting need for intervention [9].

Haemorrhage control with tourniquets has been used for centuries, but its use in both military and civilian practice has been controversial. However, evidence arising from literature in recent years has placed tourniquets in a favourable light. Schragner et al. [10] stated that there are demonstrable benefits in using tourniquets to stem haemorrhage, in lessons learnt in Iraq and Afghanistan. Whilst the use of limb tourniquet for haemorrhage control in the civilian setting remains controversial, in military series, they have been shown to contribute to a reduction in mortality especially if applied early prior to onset of refractory shock [11]. Lessons learnt in military practice have greatly influenced emergency civilian care. The application of tourniquets on civilians by emergency crew has been practiced. When tourniquets are not appropriate due to the anatomy of injury, direct pressure over the bleeding point or over the artery proximal to injury can be used as an adjunct to stem bleeding. A study on penetrating injury in the civilian population demonstrated that 57% of the study could have had an application of a tourniquet to prevent exsanguination [12]. In many regions, including our own, specialist vascular and trauma services have centralised to large urban hubs, and when damage-control skills are not locally available, rapid transport of the unstable and bleeding patient can be assisted by tourniquet use as demonstrated in our series (case 3). From experiences by the American Military, the combination of aggressive haemorrhage control and rapid transfer has shown improvement in morbidity and mortality [13]. Timely transfer to an appropriate unit where skilled multidisciplinary members can provide definitive care is vital to life and limb salvage. Trauma Team (vascular/general surgeons; orthopaedic surgeons, emergency physicians and anesthetists) can provide rapid diagnosis, goal directed resuscitation, and damage-control surgery. The experienced multidisciplinary team members work together to provide the best treatment for these trauma patients. In some setting where the extent of injury to the limb or haemodynamic instability of the patient makes attempts at limb salvage futile, the Mangled Extremity Scoring System (MESS) are useful in assisting decision-making. One retrospective study by Kumar et al. [14] demonstrated that MESS could predict amputation of severely injured lower limbs (that scored 7 or more) with 91% sensitivity and 98% specificity.

The principles of vascular trauma care should be applied when major vascular injuries are identified, as previously described, when required, in an escalating fashion from simple arrest of haemorrhage, through simple repair, to complex repair, or damage-control surgery with deferred definitive repair [2–4]. Treatment may include standard surgical approaches, but also increasingly, one should consider minimally invasive endovascular options, in particular for patients who are unstable, elderly or unfit, and to treat anatomically remote regions (chest and abdomen). Endovascular options may include the use of covered-stents or coil-embolization of bleeding vessels. Simple surgical repair can be in the form of ligation, direct suture repair, or patch repair as performed in case 3. Bypass procedures may be required for more complex injuries and autologous vein (long saphenous vein) conduits are preferred in contaminated fields or when distal bypasses are required [2]. This was demonstrated by case 2, as he underwent a popliteal to TP trunk bypass. Intraluminal shunts can be used intraoperatively to temporarily restore flow to distal extremities [2]. Their use is recommended in complex cases to allow time for reconstruction, and crucial in critical arterial territories which were distal end-organ perfusion is essential, such as in the carotid artery to protect the brain. It has been suggested that the selective use of shunts in vascular injuries of the limb may reduce amputation rates [15]. In our series, shunts have been used which allowed restoration of distal blood flow, whilst conduit vein was harvested for definitive arterial repair and also permitted time for fracture fixation prior to definitive arterial repair [2]. In military series, temporary vascular shunts have been used in damage-control surgery to restore arterial continuity during transport or whilst awaiting definitive surgical repair [13, 15]. We encourage a multidisciplinary team approach to the management of these injuries with teams often involving vascular, general, orthopaedic, and plastic surgeons. Aftercare of the patient is also essential with physiotherapy to heal the body and perhaps psychotherapy to heal the mind.

## Conclusion

Punishment shootings in Northern Ireland are once again following an upward trend. In the last decade, we have witnessed a re-emergence of punishment shootings which have evolved beyond knee-capping, to a form of anatomically targeted arterial and skeletal trauma: the phenomenon we describe here as the BLAST. These potentially devastating injuries can be managed by a multidisciplinary team experienced in vascular trauma care to achieve limb salvage and good functional outcomes.
